# Awareness of Cognitive Decline in Patients With Alzheimer's Disease: A Systematic Review and Meta-Analysis

**DOI:** 10.3389/fnagi.2021.697234

**Published:** 2021-08-03

**Authors:** Federica Cacciamani, Marion Houot, Geoffroy Gagliardi, Bruno Dubois, Sietske Sikkes, Gonzalo Sánchez-Benavides, Elena Denicolò, José Luis Molinuevo, Patrizia Vannini, Stéphane Epelbaum

**Affiliations:** ^1^Institut du Cerveau, ICM, Hôpital de la Pitié-Salpêtrière, Paris, France; ^2^Inserm, U 1127, Paris, France; ^3^CNRS, UMR 7225, Paris, France; ^4^Sorbonne Université, Paris, France; ^5^Inria, ARAMIS-Project Team, Paris, France; ^6^Institute of Memory and Alzheimer's Disease (IM2A), Centre of Excellence of Neurodegenerative Disease (CoEN), ICM, CIC Neurosciences, AP-HP, Department of Neurology, Hôpital de la Pitié-Salpêtrière, Sorbonne University, Paris, France; ^7^Department of Neurology, Massachusetts General Hospital and Harvard Medical School, Boston, MA, United States; ^8^Department of Neurology, Center for Alzheimer Research and Treatment, Brigham and Women's Hospital and Harvard Medical School, Boston, MA, United States; ^9^Department of Neurology, Alzheimer Center Amsterdam, Amsterdam Neuroscience, Amsterdam University Medical Center (UMC), Vrije Universiteit Amsterdam, Amsterdam, Netherlands; ^10^BarcelonaBeta Brain Research Center (BBRC), Pasqual Maragall Foundation, Barcelona, Spain; ^11^Hospital del Mar Medical Research Institute (IMIM), Barcelona, Spain; ^12^Centro de Investigación Biomédica en Red de Fragilidad y Envejecimiento Saludable (CIBERFES), Madrid, Spain; ^13^Department of Biomedical Science and Neuromotor Sciences, University of Bologna, Bologna, Italy

**Keywords:** awareness, anosognosia, metamemory, hypernosognosia, Alzheimer's disease

## Abstract

**Background:** Identifying a poor degree of awareness of cognitive decline (ACD) could represent an early indicator of Alzheimer's disease (AD).

**Objectives:** (1) to understand whether there is evidence of poor ACD in the pre-dementia stages of AD; (2) to summarize the main findings obtained investigating ACD in AD; (3) to propose a conceptual framework.

**Data Sources:** We searched Scopus, Pubmed, and the reference lists for studies published up to August 2020. Original research articles must report a measure of ACD and included individuals with AD dementia, or prodromal AD (or MCI), or being at risk for AD.

**Data Synthesis:** All studies covering preclinical, prodromal, and AD dementia were systematically reviewed. We intended to perform a meta-analysis of empirical studies on preclinical AD or prodromal AD (or MCI), to compare ACD between clinical groups. Due to the paucity of literature on preclinical AD, meta-analysis was only possible for prodromal AD (or MCI) studies.

**Results:** We systematically reviewed 283 articles, and conducted a meta-analysis of 18 articles on prodromal AD (or MCI), showing that ACD was not significantly different between patients with amnestic and non-amnestic MCI (SMD = 0.09, *p* = 0.574); ACD was significantly poorer in amnestic MCI (SMD = −0.56, *p* = 0.001) and mild AD (SMD = −1.39, *p* < 0.001) than in controls; ACD was also significantly poorer in mild AD than in amnestic MCI (SMD = −0.75, *p* < 0.001), as well as poorer than in non-amnestic MCI (SMD = −1.00, *p* < 0.001). We also discuss key findings on ACD in AD, such as its neural and cognitive correlates.

**Conclusions and Implications:** We propose that patients may be complaining of their initial subtle cognitive changes, but ACD would soon start to decrease. The individual would show mild anosognosia in the MCI stage, and severe anosognosia in dementia. The evaluation of ACD (comparing self-report to cognitive scores or to informant-report) could be useful to guide the clinician toward a timely diagnosis, and in trials targeting early-stage AD.

## Introduction

### Rationale

Over the past two decades, advances in basic and clinical research have provided a better understanding of the natural history of Alzheimer's disease (AD). It is now well-known that years pass before pathophysiological changes (such as the buildup of amyloid plaques and neurofibrillary tangles) lead to cognitive impairment. AD has therefore been reconceptualized as a continuum comprising three phases (Dubois et al., 2010): (i) an initial *preclinical* phase, in which the patient may show a subtle decrease in cognitive efficiency compared to his or her own baseline level, without having normal cognitive scores (*transitional cognitive decline* according to Jack et al., 2018); (ii) an intermediate phase known as *prodromal AD*, during which the patient has mild objective cognitive impairment (MCI) which does not limit his or her autonomy in daily life; and (iii) the term *dementia* indicates the final phase of the disease in which the disorders are more severe, widespread in multiple cognitive domains, and interfering with autonomy. In a recent study (Vermunt et al., 2019), the preclinical phase lasted on average 10 years, the prodromal phase 4 years, and the dementia phase 6 years, in individuals who presented with preclinical AD at 70 years of age.

The search for strategies for timely diagnosis (before patients cross the threshold of dementia) has become one of the key themes in AD research. A timely diagnosis opens up a wide spectrum of possibilities for the patients and their family, but also at the community and societal level, mainly in terms of treatment, decision-making and cost reduction (see Dubois et al., 2016b for a review). The importance of timely diagnosis is underlined by campaigns aimed at the general population, for example within the WHO Global Action Plan on dementia 2017–2025. Their goal is to promote an accurate understanding of AD, increase public knowledge about risk factors, and educate people to recognize early symptoms of AD. This is changing people's attitudes toward the disease (Cations et al., 2018, PLoS ONE).

As a result of this ongoing cultural shift, people are increasingly seeking medical advice for a self-perceived decline in cognition. A growing number of studies are currently investigating whether Subjective Cognitive Decline (SCD) could represent an early (mostly preclinical) indicator of AD. SCD is defined as a self-experienced persistent decline in cognitive capacity in comparison with a previous normal status and unrelated to an acute event, while age-, gender-, and education-adjusted performance on standardized tests is normal (Jessen et al., 2014). The idea that seems to prevail is that the expression of cognitive complaints can represent the first manifestation of AD prior to objective cognitive impairment. Results are rather conflicting but various studies have identified an increased likelihood of biomarker abnormalities consistent with AD pathology in individuals with SCD (e.g., ApoE ε4 allele overrepresentation in Abdulrab and Heun, 2008; abnormal amyloid levels in Wolfsgruber et al., 2015; regional hypometabolism in Mosconi et al., 2008; atrophy in Garcia-Ptacek et al., 2014). According to the most recent criteria of SCD (Jessen et al., 2020), individuals aged 60 years or over, persistently worried by a memory decline for at least 5 years, for which they have sought medical advice, and which is confirmed by an informant, would be more at risk of preclinical AD.

These criteria for SCD are still subject to ongoing validation and refinement, as the authors also stated (Jessen et al., 2020). Some studies in recent years have attempted to go further in describing how patients with early-stage AD experience their progressive cognitive decline. It has recently been proposed that exhibiting a poor awareness of cognitive decline (ACD) could represent an early clinical indicator of the disease (Cacciamani et al., 2017), more specific than SCD, and should encourage more in-depth patient monitoring. The lack of awareness of illness is indeed a known symptom of AD, especially in the dementia phase, in which it goes under the name of *anosognosia*. The term *anosognosia* derives from the Greek α (without), νoσoζ (disease or illness), γνωσ*ι*ζ (knowledge) (Babinski, 1914, translated by Langer and Levine, 2014).

According to the Dissociable Interactions and Conscious Experience (DICE) theory (McGlynn and Schacter, 1989; Schacter, 1989; McGlynn and Kaszniak, 1991), the activation distinctive cognitive modules representing specific cognitive functions would trigger the Conscious Awareness System, resulting in conscious awareness of the information being processed. Damage to one or more individual modules, or their disconnection from the Conscious Awareness System, due to brain damage, would result in a domain-specific deficit in awareness. The Conscious Awareness System itself could be damaged, resulting in a generalized unawareness. Agnew and Morris (1998) and Mograbi et al. (2009) extended the DICE model, which was renamed *Cognitive Awareness Model*. According to this new model, the Conscious Awareness System, which would be located in the parietal lobe, processes the feedback received after an action has been executed: in this way, the individual becomes aware of having performed it correctly or not. Then, the mnemonic comparator, located within the executive system, would compare this knowledge with existing information about the individual's abilities. If it does not match with the semantic personal knowledge base, this latter would be updated. Anosognosia in AD dementia has been suggested to arise from a suboptimal ability to detect a mismatch between current performance and past knowledge about the self, and to the inability to recollect and update personal semantics (Graham et al., 2005). Mograbi et al. (2009) added that AD mainly affects recent memories and predominantly spares older information about the self, since the oldest memories are located in the neocortex and therefore less dependent on hippocampus integrity. This amnestic pattern, together with executive dysfunction, would result in a *petrified* self-evaluation based on premorbid abilities (Kalenzaga and Clarys, 2013). Recent studies have provided partial support to the *Petrified-self* theory. Patients with AD dementia may acknowledge their deficient performance shortly after its execution, and use this information to partially and temporarily update their self-knowledge. However, this new knowledge about the self fails to be used and integrated into long-term self-representations (Gil and Josman, 2001; Duke et al., 2002; Ansell and Bucks, 2006; Mimura and Yano, 2006; Hannesdottir and Morris, 2007; Oyebode et al., 2007; Stewart et al., 2010; Silva et al., 2017; Bertrand et al., 2019).

The possibility that poor ACD could serve as an early indicator of AD may seem to run counter to research results on SCD. However, the concepts of *SCD* and *poor awareness* are only apparently opposed since they can coexist in the same individual, as found in the INSIGHT-PreAD cohort (Cacciamani et al., 2017, 2020). This is the case of individuals who complain of a certain degree of cognitive difficulties, still underestimating their severity or impact on daily life (when compared to the assessment made by an informant or using cognitive tests). Studying the degree of ACD in AD continuum helps us to better understand how patients experience the disease, and therefore better characterize the cognitive complaints typical of the patient with AD.

The methods commonly used to assess ACD in the context of AD in research and clinical settings can be categorized as follows.

The first category includes the evaluation of the clinician, who asks the patient more or less structured questions about the reason for the visit or whether he or she perceives cognitive difficulties (e.g., Cova et al., 2017). This is a time-saving method, but its psychometric robustness is not always known.

A second category is performance-based methods, assessing (i) the discrepancy between objective cognition and self-reported cognition (Dalla Barba et al., 2015); and (ii) the accuracy of pre-test predictions or post-test estimates of performance (Graham et al., 2005; Hannesdottir and Morris, 2007; Mograbi et al., 2012). Hannesdottir and Morris for example propose Objective Judgment Discrepancy to measure awareness of memory performance (or memory-monitoring). The clinician or investigator asks the individual to estimate the number of successfully remembered items in a memory test, and then applies the following formula: [(estimated score-actual score) / maximum possible score on measure] × 100. The main difficulty related to these methods is that it could be challenging for an individual to evaluate the performance on unfamiliar cognitive tasks.

The third category of methods includes the discrepancy between the cognitive difficulties perceived by the patient and those reported by an informant (a family member or close friend). This is generally calculated by asking the patient and an informant to separately fill in parallel forms of the same questionnaire that assesses the patient's cognitive functioning (e.g., Edmonds et al., 2018). The discrepancy between these two scores can be treated as a continuum, or a cut-off can be identified to attribute an awareness status to the subject. We describe here the main questionnaires allowing to compute the subject-informant discrepancy. The *Cognitive Change Index* (CCI, Rattanabannakit et al., 2016) is a widely used questionnaire. Two parallel forms are available (one for the patient and one for an informant), in which they assess the severity of recent changes in memory (12 items), in attention and executive functions (5 items), and in language (3 items). The *Everyday Cognition Questionnaire* (Farias et al., 2008), known as E-Cog and also used in the Alzheimer's Disease Neuroimaging Initiative (ADNI) cohort study (http://adni.loni.usc.edu), asks the patient and an informant to evaluate how much specific domains have changed compared to 10 years ago: everyday memory, language, visuospatial abilities, planning, organization, and divided attention. Another questionnaire used in the literature and of less recent construction is the *Anosognosia Questionnaire-Dementia* (AQ-D) by Migliorelli et al. (1995). It is a 30-question questionnaire that assesses the frequency of cognitive, functional and behavioral changes. The *Healthy Aging Brain Care (HABC) Monitor* is a valid and reliable tool to compare the self- and informant-report of decline. It includes questions relating to the cognitive, functional and psycho-behavioral spheres (Monahan et al., 2012, 2014). Finally, the *Patient Competency Rating Scale* (PCRS) was developed by Prigatano (1987) to evaluate anosognosia following brain trauma. It includes 30 questions covering cognitive, but also behavioral and functional domains. The patient and a person who knows him/her well (a family member or a clinician) use a 5-point Likert scale to assess the degree of difficulty in the aforementioned contexts.

It has two parallel forms for the patient and an informant, thus allowing to calculate the discrepancy between the two reports. The questions ask to assess the frequency of 30 cognitive difficulties or behavioral changes. The subject-informant discrepancy is one of the most used methods in literature to measure ACD. However, few studies explored the psychometric properties of these questionnaires (e.g., Gil and Josman, 2001; Monahan et al., 2012, 2014). The subject-informant discrepancy method assumes that the informant's report is an accurate source of information. However, the possibility that the informant's report could be distorted by factors such as anxiety, depression, burden, personality traits, should also to be taken into consideration. In Cacchione et al. (2003), for instance, the informant's rating significantly predicted his or her actual cognitive decline, and its accuracy was above case even for informants who were not spouses, who did not live with the patient, or who spoke to the patient less than daily, and for patients who were older or less educated.

See Rabin et al. (2015) for a review.

### Objectives

We aimed at providing a synthesis of the current state of the art of scientific literature investigating ACD in relation to AD. Qualitative and quantitative methods have been used to describe ACD in AD, to (1) understand whether there is evidence of poor ACD in the pre-dementia stages, and therefore whether it can be used as an early indicator of AD; (2) qualitatively summarize the main results obtained for a better understanding the neural and clinical correlates of ACD in the different stages of the disease; (3) outline a theoretical framework, useful in clinical practice in the context of early AD diagnosis and in research to motivate further studies and to suggest where future research might be best directed.

## Methods

### Protocol and Registration

The review and meta-analysis protocol have not been published elsewhere than in this article.

### Search Strategy and Study Eligibility Criteria

Studies were identified by searching two electronic databases (PubMed and Scopus). The reference list of the resulting articles was also hand-searched to find additional relevant articles.

Search terms were: “(*Alzheimer disease OR Mild Cognitive Impairment) AND (awareness OR metacognition OR anosognosia*)” (MeSH terms when relevant). We imposed no restrictions in terms of study type (we included original research papers, reviews and meta-analyses) and publication date. In fact, we wanted to include all eligible articles published until August 2020, when the literature search was carried out.

Original research articles must report at least one measure of ACD. Review articles must discuss ACD or anosognosia in relation to AD. Studies that exclusively addressed the awareness of non-cognitive changes (for example, awareness of functional decline, or psycho-behavioral disorders) were excluded.

For the selection of articles, we have taken into account that many diagnostic labels have been proposed over the years and that preclinical AD is a newly formulated concept. Therefore, we have established that subjects must be classified as: (i) cognitively-intact at risk for AD including at least one biomarker for AD and the findings discussed within the scope of preclinical AD; or (ii) subjects classified as having a MCI, with or without *in vivo* evidence of AD pathology, with no restrictions in terms of diagnostic criteria used or type of MCI (e.g., amnestic or non-amnestic); or (iii) individuals diagnosed with AD dementia, regardless of the diagnostic criteria used. We imposed no demographical restrictions.

Articles must be in English or French.

### Study Selection

Two authors (FC and GG) reviewed all retrieved records by reading the title and abstract and, if necessary, the body of the article. We checked whether the articles met the eligibility criteria and issued a decision independent of each other. In the case of ineligibility, they recorded the reason. Subsequently, they discussed to reach a common agreement for each article. None of the authors were blind to the study authors, their affiliations, or journal title.

### Data Collection Process and Data Items

For all original research articles, we used an uncoded form, along the lines of the Cochrane Data Extraction Form. We pilot-tested it on five randomly-selected studies, and no refinement was needed. For each of these studies, we recorded: (a) aim, (b) sample size, (c) diagnostic classification of the participants, (d) mean age, (e) mean and (f) range of the Mini-Mental State Examination (MMSE) when available, (g) mean years of education, (h) percentage of men, (i) measure used to assess ACD, (j) statistical model performed, and (k) key findings relevant for this review.

We also had additional coded items, to be filled in only if the original research article included at least a subgroup of subjects at risk for AD (or with preclinical AD) or prodromal AD (or MCI), as we decided to perform a meta-analysis (Objective 1). Relevant information to be extracted was determined a priori as follows:

*1: Studies treating the measure of ACD as a continuous variable*. We extracted (l) mean ACD of each clinical group (i.e., cognitively-normal, amnestic MCI, non-amnestic MCI, mild dementia, moderate dementia, severe dementia), (m) standard deviation (SD) for each clinical group, (n) size of the whole sample, and (o) size of each clinical group being compared in the study. When relevant, continuous measures of ACD were multiplied by −1 so that, for each article, a lower value represented a poorer ACD, and a higher value a higher ACD.

*2: Studies treating the measure of ACD as a categorical variable*. We extracted the (p) percentage of subjects with impaired ACD of each clinical group, (q) size of the whole sample, and (r) size of each clinical group being compared in the study. In particular, we considered the ACD as impaired when classified by the authors as both shallow or completely lacking, according to an established threshold.

The studies on preclinical AD and on prodromal AD (or MCI) that reported neither mean ACD (and SD) nor percentages of subjects with impaired ACD were systematically reviewed but excluded from the meta-analysis.

In the meta-analysis, the indices of ACD were considered as comparable, even if measured with different methodological approaches.

Regarding past literature reviews, we used a separate uncoded form to extract (a) the number of studies included, (b) search strategy, (c) stage of the disease, and (d) key results.

Data extraction was carried out by three authors (FC and GG independently, then MH for verification of coded items). Any discrepancies between the authors were resolved by discussion.

### Planned Methods of Analysis

The review of these articles will be addressed thematically by stage of the disease, which means that we will discuss the degree of awareness of patients in dementia, prodromal (or MCI), and preclinical stages, separately.

We intended to systematically review all the articles and to conduct a meta-analysis only of those including at least a subgroup of subjects at risk of AD (or with preclinical AD), or with prodromal AD (or MCI). Indeed, we wanted to place special emphasis on the degree of ACD in the pre-dementia stages, which is currently being debated, although we included studies on all three stages to investigate ACD throughout the entire course of AD.

We decided a priori that we would conduct a meta-analysis when at least three articles compared the same pair of clinical groups.

### Summary Measures

A random-effect meta-analysis using the inverse variance method was performed for each pairwise comparison. For articles treating the measure of ACD as a continuous variable: we estimated a standardized mean difference (SMD) between clinical groups using Hedges' g method. For articles treating the measure of ACD as a categorical variable: we estimated the odds ratio (Robins et al., 1986) and converted it to Hedges' g estimate (see Borenstein et al., 2009) in order to make these studies comparable to those that treated ACD as a continuous variable.

Heterogeneity was tested using Cochran's Q test and assessed through I^2^ and Tau^2^ indexes.

Statistical analyses were performed using R 3.6.1. and the meta (V. 4.9-7) package.

### Assessment of the Risk of Bias

To ascertain the validity of the included studies, we a priori identified some potential risks of bias and noted them when extracting data from the studies: (i) heterogeneity of study populations (e.g., in terms of age, sex, education); (ii) unclear stage of disease (e.g., inclusion of subjects with a diagnosis of AD dementia without specifying stage of severity); (iii) absence of evidence of abnormal AD biomarkers in case of MCI diagnosis; (iv) heterogeneity in the definition of preclinical AD.

## Results

### Study Selection

A flow chart showing the selection process and results is provided as Supplementary Materials. The bibliographical search yielded 662 citations, published between 1991 and August 2020. Of these, 379 did not meet the eligibility criteria and were excluded. Two hundred and eighty-two articles were systematically reviewed.

**Figure 1:**
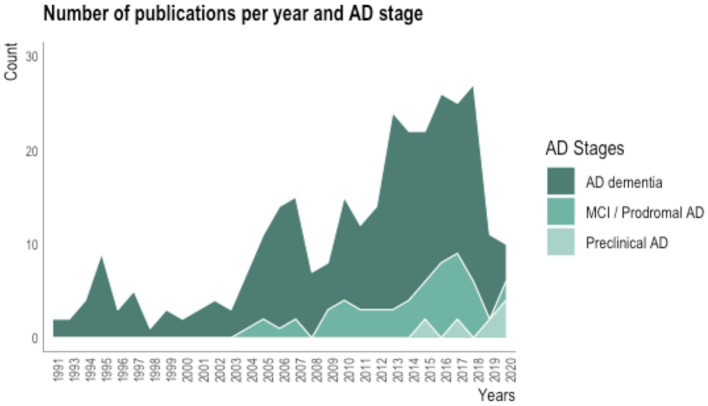
Plot showing the number of revised publications about awareness of deficits in AD per year and stage of the disease.

Figure 1 shows the number of revised publications per year and stage of the disease addressed.

We systematically reviewed 52 studies including subjects with prodromal AD or MCI. Eighteen of these were eligible for the meta-analysis, as they reported either a mean index of ACD (and SD) or the percentage of subjects with impaired ACD (see Supplementary Table 1 for more details). These studies compared ACD between clinical groups: controls, amnestic MCI, non-amnestic MCI, mild AD. The group of participants with moderate AD was excluded from the meta-analyses, as they were only compared with subjects with amnestic MCI in 1 article and with mild AD in 1 article.

On the contrary, the meta-analysis of the studies on preclinical AD was not possible, as the articles were too few (<3 articles comparing the same clinical groups).

### Characteristics and Key Findings of Studies on AD Dementia

#### Prevalence of Anosognosia in AD Dementia

The prevalence of anosognosia in AD dementia has been estimated between 40 and 91% based on the study, this range varying according to the severity of dementia, which was found to be the main determinant of anosognosia (Akai et al., 2009; Maki et al., 2013; Turró-Garriga et al., 2016).

Prevalence estimations may also vary according to how the anosognosia was operationalized and measured. In fact, all three studies that identified a lower prevalence of anosognosia in dementia (around 40%) had used the Awareness of Deficit Questionnaire-Dementia (AQ-D; Migliorelli et al., 1995). It consists of 30 questions in which the patient and an informant assess separately the frequency of certain cognitive difficulties, difficulties in everyday tasks, and changes in interests and mood. In contrast, a higher prevalence of anosognosia in dementia was found for instance in Lacerda et al. (2020) using the Assessment Scale of Psychosocial Impact of the Diagnosis of Dementia (Dourado et al., 2007, 2014). This is a 23-question-semi-structured interview, assessing awareness in the domains of cognition, social functioning, emotional status, and activities of daily living.

#### Neural Correlates of Anosognosia in AD Dementia

Anosognosia appears to be present in those demented AD patients who have particular frontal and temporoparietal lesions.

More specifically, anosognosic patients with mild to severe AD showed reduced perfusion, glucose metabolism and gray matter volume in the prefrontal cortex (PFC), both dorsolateral and in the anterior cingulate gyrus (Harwood et al., 2005; Hanyu et al., 2008; Jedidi et al., 2014; Fujimoto et al., 2017).

Others found that anosognosia was associated with reduced intrinsic connectivity and functional changes of brain areas known to be involved with self-referential processes, such as the orbitofrontal cortex (OFC), the posterior cingulate cortex (PCC) and the medial temporal lobe. For instance, Kashiwa et al. (2005) found that anosognosic patients had reduced perfusion in the OFC, and a blood flow that was reduced in the right PFC, and increased in the left temporoparietal junction. One study by Therriault et al. (2018), found that anosognosia correlated with a greater amount of amyloid in the PCC.

In Fujimoto et al. (2017), the medial temporal lobe, which is usually damaged in AD dementia, was not associated with anosognosia. On the contrary, others found greater hypometabolism (Salmon et al., 2006) and atrophy in the medial temporal lobe (Tondelli et al., 2018) in anosognosic patients.

#### Clinical Correlates of Anosognosia in AD Dementia

Executive dysfunction is highly associated with anosognosia in patients with AD dementia (Lopez et al., 1994; Kashiwa et al., 2005; Amanzio et al., 2013). The ability to inhibit a response, “on-line” self-monitoring and set-shifting appeared to be important skills for awareness in a sample of patients with mild AD (Amanzio et al., 2013). Anosognosia was associated with both disinhibition as a psychiatric symptom (assessed using the Neuropsychiatric Inventory), and response inhibition impairment as a frontal cognitive dysfunction (Kashiwa et al., 2005).

Additionally, AD patients with the poorest memory functioning rated their performance highest (Gallo et al., 2007; Gilleen et al., 2014).

Moreover, there is compelling evidence that anosognosic AD patients report better-perceived quality of life, compared to those with normal insight (Comijs et al., 2002). It has importantly been found that depression and not awareness is the key driver of the quality of life: high ACD is only indirectly associated with lower quality of life *via* depressed mood (Risacher et al., 2016). Anosognosic patients generally show lower levels of depression and anxiety, compared to non-anosognosic patients (Horning et al., 2014).

Finally, several studies have shown that anosognosic patients, although less depressed and with better-perceived quality of life, have higher levels of apathy (Hurt et al., 2010; Trigg et al., 2011; Conde-Sala et al., 2013, 2014; Millenaar et al., 2017; Stites et al., 2017). It is known that apathy—as well as anosognosia—is related to frontal lobe dysfunction (Cines et al., 2015), thus apathy and anosognosia may be two consequences of frontal damage due to AD pathology. The reciprocal relationship between anosognosia and apathy still needs to be clarified.

### Characteristics and Key Findings of Studies on Prodromal AD or MCI

#### Prevalence of Anosognosia in Prodromal AD (or MCI): Results of the Meta-Analysis

The mean number of MCI participants enrolled in the analyzed studies was on average 76.5 [Interquartile range (IQR) = 20.50–71.00]. Mean age was on average 74.1 (IQR = 72.78–76.10). Mean years of education were on average 11.5 (IQR = 9.32–13.61). Mean percentage of men was on average 47.45 (IQR = 42.80–54.60). Mean MMSE was on average 26.8 (IQR = 26.23–27.40).

Thirteen studies assessed the ACD as the discrepancy between subject's and informant's ratings of decline (Vogel et al., 2005; Onor et al., 2006; Ries et al., 2007; Orfei et al., 2010; Galeone et al., 2011; Spalletta et al., 2012; Maki et al., 2013; Zamboni et al., 2013; Ford et al., 2014; Jacus et al., 2015; Senturk et al., 2017; Tondelli et al., 2018; Oba et al., 2019). Four studies as the discrepancy between subjective and objective scores of cognitive functioning (O'Connell et al., 2014; Coutinho et al., 2016; Vannini et al., 2017a; Hanseeuw et al., 2020). In Stites et al. (2017), participants who responded affirmatively to any of the diagnosis-related questions were classified as “aware” of their diagnosis, whereas all others were coded “unaware.”

**Figure 2:**
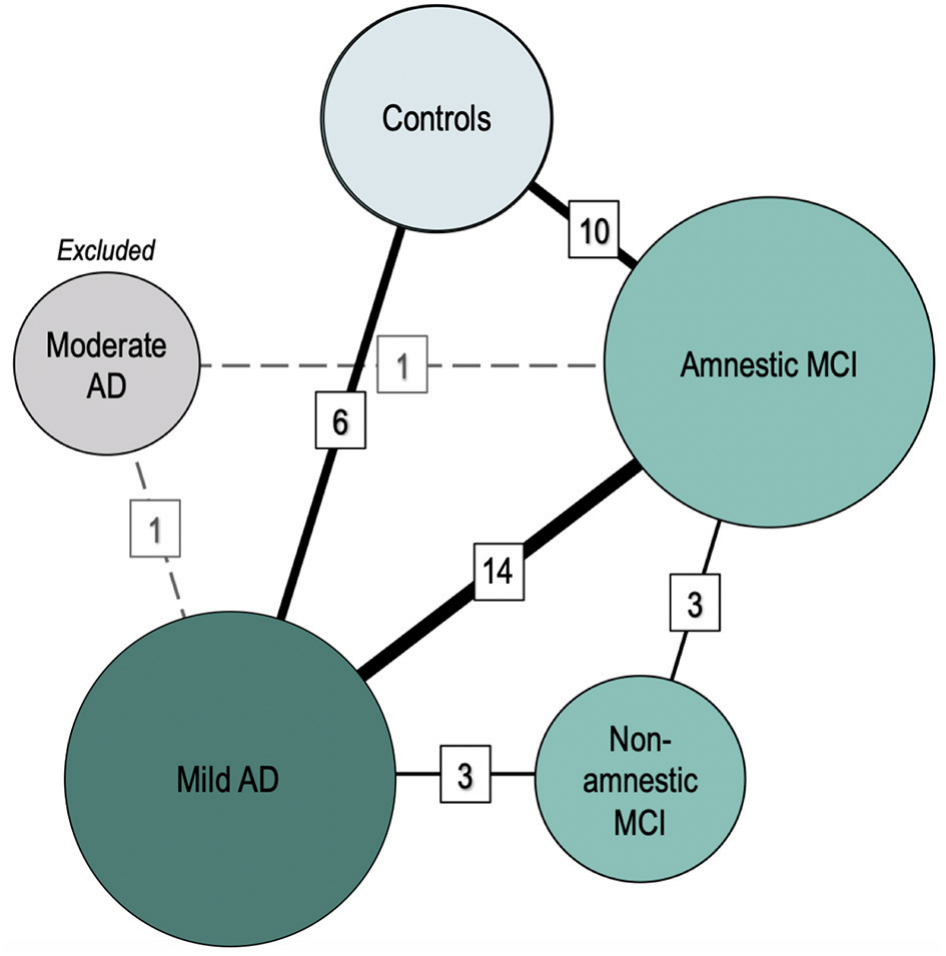
Comparisons between clinical groups (meta-analysis). Nodes represent clinical groups. The size of the nodes is proportional to the number of studies including a certain clinical group. Solid lines connect the groups that have been studied in head-to-head comparisons in the meta-analyses. Dashed lines represent non-eligible connections (number of comparisons <3). Line thickness is proportional to the number of studies performing each comparison. The numbers in cells represent the number of comparisons available between two given groups.

Figure 2 and Table 1 represent the between-group comparisons.

**Table 1:** Results of the meta-analyses comparing mean ACD between groups.

**Group comparison**	**Number of studies**	**SMD [95% CI]**	***I^**2**^***	***Tau^**2**^***	***P***	
					**Comparison**	**Heterogeneity**
Non-amnestic (*n* = 113) vs. amnestic MCI (*n* = 109)	3	0.09 [−0.21; 0.39]	20.12	0.01	0.575	0.286
Amnestic MCI (*n* = 869) vs. controls (*n* = 815)	10	−0.56 [−0.88; −0.25]	80.91	0.19	0.001^*^	<0.001^*^
Mild AD (*n* = 781) vs. amnestic MCI (*n* = 1,050)	14	−0.75 [−1.02; −0.48]	79.41	0.19	<0.001^*^	<0.001^*^
Mild AD (*n* = 226) vs. non-amnestic MCI (*n* = 113)	3	−1.00 [−1.25; −0.76]	0.00	0.00	<0.001^*^	0.887
Mild AD (*n* = 320) vs. controls (*n* = 468)	6	−1.39 [−1.92; −0.85]	84.10	0.36	<0.001^*^	<0.001^*^

*SMD, Standardized mean difference; CI, Confidence interval. n: Pooled group size. The I^2^ statistic describes the percentage of variation across studies that is due to heterogeneity rather than chance. Tau^2^ indicates the extent of variation among the effects observed in different studies (between-study variance)*.

* *Statistically significant at the 0.01 level*.

Forest plots are included as Supplementary Figure 1.

The degree of ACD was not significantly different between patients with amnestic and non-amnestic MCI [SMD (95% CI) = 0.09 (−0.21; 0.39), *p* = 0.574]. On average, the ACD was significantly lower in amnestic MCI [SMD (95% CI) = −0.56 (−0.88; −0.25), *p* = 0.001] and in mild AD [SMD (95% CI) = −1.39 (−1.92; −0.85), *p* < 0.001] than in controls. ACD was also significantly poorer in mild AD than in amnestic MCI [SMD (95% CI) = −0.75 (−1.02; −0.48), *p* < 0.001], as well as poorer than in non-amnestic MCI [SMD (95% CI) = −1.00 (−1.25; −0.76), *p* < 0.001].

The articles comparing subjects with non-amnestic vs. amnestic MCI had low heterogeneity (I^2^ = 20%; Tau^2^ = 0.01, *p* = 0.286), as well as those comparing subjects with mild AD vs. non-amnestic MCI (I^2^ = 0%; Tau^2^ = 0.00, *p* = 0.887). On the contrary, heterogeneity of articles performing all other comparisons was substantial and significative (all I^2^ > 79%; all Tau^2^ = 0.36; all *p* ≤ 0.001).

#### Neural Correlates of Anosognosia in MCI

Few studies have investigated the neural correlates of ACD in MCI, indicating an involvement of a set of frontal and temporoparietal regions. This would be consistent with what has been identified in the studies including participants with AD dementia. In Ries et al. (2007), for instance, MCI participants showed subtly attenuated cortical midline structures activity during a fMRI self-appraisal task. They also found that poor ACD was significantly associated with attenuated activation in PFC and PCC during self-appraisal. In a study by Nobili et al. (2010), the PCC, the inferior parietal lobe, the angular gyrus and the precuneus seemed to be a key node of the network being involved in ACD. Similarly, Vannini et al. (2017b) found that the participants with amnestic MCI who showed greater anosognosia had a reduced glucose metabolism in the PCC and the hippocampus, and increased intrinsic connectivity disruption between the PCC and the orbitofrontal cortex as well as between the PCC and the inferior parietal lobe.

Tondelli et al. (2018) studied the neuroanatomical correlates of the three most commonly used methods to assess anosognosia (i.e., clinician rating, participant-informant discrepancy and subjective-objective discrepancy) in a sample of amnestic MCI patients and healthy controls. They found that all three scores positively correlated with atrophy in the medial temporal lobe, including the right hippocampus.

#### Clinical Correlates of Anosognosia in MCI

In the study of Senturk et al. (2017), ACD positively correlated with MMSE and episodic memory, working memory, and executive functions scores. In Tremont and Alosco (2011), the anosognosic patients were comparable to non-anosognosic ones in all demographic characteristics, cognitive and behavioral domains, except that anosognosic patients obtained significantly lower scores in the learning domain.

In the study of Vogel et al. (2005), anosognosia positively correlated with cognitive impairment (MMSE score) and right inferior frontal gyrus blood flow, but not to tests of executive functions, both in MCI and AD dementia patients.

Furthermore, some authors have suggested that anosognosia in MCI is more related to non-cognitive (i.e., psychiatric) factors. In Oba et al. (2019), those who had no depressive symptoms were able to more accurately evaluate their memory impairment, suggesting that anosognosia should not be considered as a specific symptom of AD but as the result of an interaction between memory impairment and depression. Jacus et al. (2015) found a negative correlation between the degree of ACD and apathy.

#### Anosognosia in MCI and Risk of Progression to Dementia

The presence of anosognosia in a patient with MCI seems to increase the risk that he or she is affected by AD. A recent 2-year longitudinal study (Therriault et al., 2018) found that anosognosic MCI participants showed greater amyloid burden and reduced brain metabolism in the posterior cingulate cortex at baseline than those without anosognosia, and had 3 times the risk of progression to dementia after 2 years. Furthermore, anosognosia at baseline predicted a reduced metabolism in the default mode network at the follow-up. Another 2-year long longitudinal study (Edmonds et al., 2018) also showed progressive anosognosia through the stages of MCI and dementia, driven by an increase in informant-reported ratings, despite stable self-reported complaints. In this study, anosognosic MCI participants had higher rates of cerebrospinal fluid AD biomarker positivity and progression to dementia.

Similar results have been reported in Munro et al. (2018) and Scherling et al. (2016).

In contrast with these studies, few others have found that the predictive value of reduced ACD was low. Cova et al. (2017), for example, found no relationship between ACD and progression to AD dementia after 28 months, but the authors commented on their result in light of a possible inadequacy of the method to measure anosognosia (a single question from the Geriatric Depression Scale being too simple a way to measure a complex symptom such as anosognosia).

It must be noted that in these studies MCI was seen as a possible transition phase between normal cognition and AD dementia, most of them did not include biomarker evidence to support AD pathology, thus questioning the appropriateness of the conclusions drawn regarding MCI due to AD (or prodromal AD). Indeed, MCI is a heterogeneous clinical entity, possibly resulting from different etiologies (e.g., neurodegenerative diseases, vascular lesions, psychiatric disorders, non-neurological diseases, among others) and with different clinical pictures and courses (declining, stable, or reversible).

### Characteristics and Key Findings of Studies on Preclinical AD

Up until August 2020, 8 studies investigated ACD in asymptomatic individuals at risk for AD, and discussed the results within the scope of preclinical AD.

The first study that proposed the reduction of ACD as a more specific indicator of early-stage Alzheimer's than SCD is Cacciamani et al. (2017), investigating a cohort of memory-complainers at risk of preclinical AD due to their age and positive amyloid PET scan in 30% of subjects. Nineteen participants were found to have poor ACD, meaning that despite complaining about their memory, they reported less difficulty than their study-partner. This group was compared to 86 participants with heightened ACD, i.e., reporting more cognitive difficulties than their study-partner. The low ACD group had greater amyloid deposition than those with heightened ACD. Forty-seven percentage of subjects with low ACD were amyloid positive, vs. 24% of those with heightened ACD. The participants with low ACD also had a lower cortical glucose metabolism in frontal and temporoparietal regions known to be involved in both AD and anosognosia. On the contrary, the measures of SCD alone, i.e., without comparison with the informant report, did not correlate with any AD biomarker. Similarly, Sanchez-Benavides et al. (2018) compared the level of anxiety and depression, cognitive performance and brain atrophy of three groups of individuals from the ALFA cohort (Molinuevo et al., 2016): informant complaint only (therefore unaware subjects), subjects with SCD (with or without informant complaints) and controls (neither the subject nor the informant reported a decline). SCD subjects reported greater anxiety and depression than both unaware subjects and controls. Unaware subjects showed a poorer memory performance than controls (but no differences compared to SCD) which correlated with lower left posterior hippocampal volume. Unaware subjects presented brain volume increments in self-appraisal areas (medial frontal and insula). For this latter finding, they hypothesized non-linear volumetric changes, in which the volume of gray matter would increase and then decrease.

In two cross-sectional studies, ACD was non-linearly associated with amyloid load. In Vannini et al. (2017a), whereas cognitively-intact subjects harboring amyloid pathology at PET presented with *hypernosognosia* (self-report > informant-report), MCI patients with increased amyloid pathology showed anosognosia. In contrast, MCI patients with low amounts of amyloid were observed to have normal insight. Altered ACD tracked with amyloid pathology. A similar non-linear association was observed in Gagliardi et al. (2020) in 448 cognitively-normal individuals with SCD from the INSIGHT-PreAD and ADNI cohorts. ACD increased with increasing amyloid load up to a certain point, above which the increase in amyloid load was associated with a decline in ACD. Interestingly, the inflection point was around the amyloid positivity threshold, suggesting that complaints progress into decreasing ACD when the participants become amyloid-positive. In this study, the authors introduced and validated the Meta-Memory Ratio (MMR), a cohort independent measure of ACD based on the discrepancy between subjective and objective measures of cognitive decline.

Verfaillie et al. (2019) found different results by studying 106 SCD memory-clinic patients with amyloid PET scans from the Subjective Cognitive ImpairmENt Cohort (SCIENCe) study (Slot et al., 2018). They used two measures of ACD: (1) self-reported Cognitive Change Index (CCI) minus episodic memory; (2) a self-proxy index (self- minus informant-reported CCI). In this study as well as in the previous ones, amyloid burden was more associated with ACD than with self-report alone. However, amyloid burden was associated with heightened and not reduced ACD, and only when ACD was measured as a subjective-objective memory scores discrepancy. Significant interaction with education was found, implying a stronger effect in those with lower levels of education. These findings underline the fact that demographic features might be of importance when studying ACD.

To our knowledge, the first longitudinal study investigating ACD in cognitively-normal and MCI subjects at baseline was Wilson et al. (2015). A composite measure of memory performance was regressed on memory rating (i.e., two questions about their memory). In the subset of participants who progressed to dementia (*n* = 239), ACD was stable up to 2.6 years before dementia. During the prodromal phase, the ACD began to decline rapidly. This implies that the subjects had normal insight for the duration of the preclinical phase. However, in two more recent studies using more advanced statistical methods, ACD began to decline already in the preclinical phase, leading to anosognosia in the prodromal phase. Hanseeuw et al. (2020) studied the ADNI cohort and specifically amyloid-positive and amyloid-negative subjects with normal cognition, MCI and dementia. They computed the subject-informant discrepancy on the Everyday Cognition scale (ECog—memory subscale). ACD persistently declines across disease progression (controls > MCI > AD). The decline in ACD was driven by increasing study-partners' ratings over time and stable patients' ratings. It decreased faster in amyloid-positive participants. The interaction between amyloid load and clinical group had a significant effect on ACD changes in dementia and MCI groups, and had a small but significant effect also in CN subjects, suggesting that ACD starts to decrease in the preclinical AD stage. Cognitively-normal subjects reported significantly more cognitive complaints than their study-partners up to 1.6 years before progression to MCI indicating a state of heightened ACD (or hypernosognosia). Anosognosia was observed in individuals with MCI 3.2 years before progression to dementia. Low ACD predicted a greater risk of subsequent progression to dementia in participants with MCI as well as CN individuals with equal amyloid load and memory performance. Both the participants with low amyloid load and their study-partners reported more difficulties over time, resulting in stable ACD. A second longitudinal study (Vannini et al., 2020) selected 396 presenilin (PSEN1 E280A) variant carriers from the Colombia Alzheimer's Prevention Initiative Registry (Tariot et al., 2018), 59 of which were cognitively-impaired. ACD was measured as the subject-informant discrepancy on a memory complaint scale (Gatchel et al., 2020). The subjects presented with heightened ACD until on average 35 years of age and had anosognosia at ~43 years of age (~6 years before their estimated median age of dementia onset).

In summary, studies on ACD in preclinical AD are still few and heterogeneous. The main problem is the diversity in subjects inclusion criteria. However, these findings encourage a more in-depth study of how aware individuals who are developing AD are about their cognitive performance. This is particularly interesting considering that preclinical patients may show some decline from their previous cognitive efficiency, even though they do not by definition have frank cognitive impairment. The prevailing idea of the aforementioned articles is that the measure of ACD (hence the discrepancy between self-report and informant-report, or between self-report and objective scores) could be a more specific indicator of future cognitive decline than self-reported complaint alone as it is often studied in literature. However, it is not yet entirely certain whether hypernosognosia or reduced nosognosia is more characteristic of preclinical AD.

## Discussion

In this review, we summarized previously published studies about awareness of cognitive decline in AD over its full spectrum.

**Figure 3:**
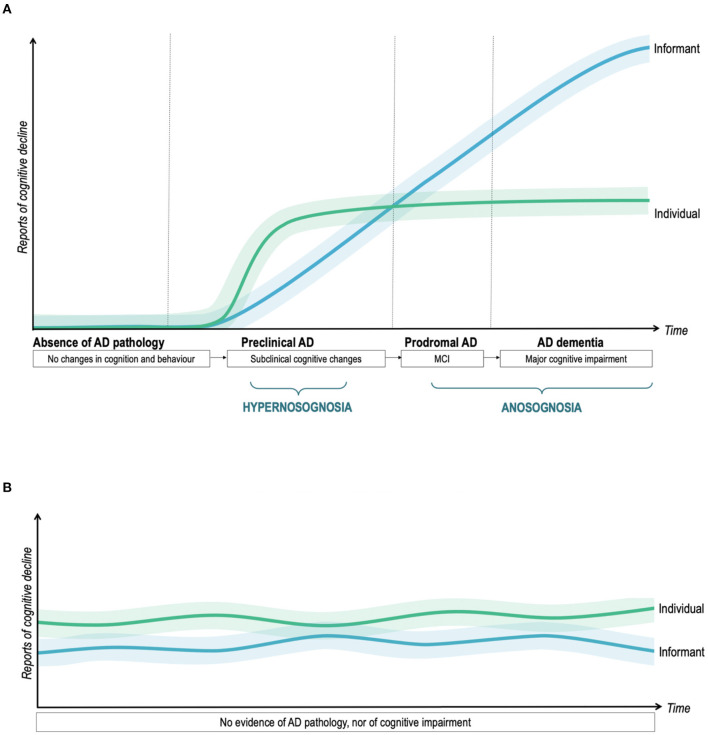
Profiles of subjective reports of decline in individuals with and without AD pathology. **(A)** ACD in the AD continuum. **(B)** ACD in the “worried-well” population. Longitudinal trajectories of ACD in AD and “worried-well” individuals, conceptualized as the discrepancy between patient- and informant-reported cognitive decline. The green line displays how the individual's rating changes over time. The blue line represents the evolution of the informant's rating. The lighter areas represent potential individual deviations from the typical trajectories. In **(A)**, the duration of the disease stages has been established with reference to Vermunt et al. (2019).

In Figure 3A, we graphed the longitudinal changes in the ACD based on the existing results.

Studies targeting preclinical AD identified an increased risk of AD (mainly abnormal biomarkers) both in subjects overestimating and underestimating their cognitive performance (when compared to their informant or to cognitive tests). Recent longitudinal studies (such as Vannini et al., 2017a; Hanseeuw et al., 2020) suggested that these two different states of altered ACD come in succession during preclinical AD in the same individual. This means that patients with very early-stage AD would begin to notice their first subtle cognitive changes when people around them do not yet, and neuropsychological tests do not yet detect objective cognitive deficits. They would therefore experience a *hypernosognosia* (in the terminology proposed by Vannini et al., 2017a) and might seek medical advice. Later, but still very early across the course of the disease, the patient's family or friends also begin to notice these changes as his or her cognitive efficiency gradually declines. The patient would soon begin to underestimate his or her impairment. The ACD is beginning to decline.

We would like to discuss a recent study on ACD in subjects at risk of preclinical AD, published after our bibliographic search (in October 2020) therefore not included in our review and meta-analysis. This study (Cacciamani et al., 2020) describes different trends of evolution of ACD over 3 years in the INSIGHT-PreAD cohort (memory-complainers, Dubois et al., 2018) and their association to amyloid burden and brain metabolism. 76.8% of the sample (235 out of 306 subjects) had an accurate ACD (i.e., self-report = informant-report), which remained unchanged over time. This class was chosen as the reference as it indicated normal insight. 18.95% (58 subjects) showed a steadily heightened ACD (i.e., self-report > informant-report). Interestingly, they were comparable to those with accurate ACD in terms of demographic characteristics and AD biomarkers, meaning that persistent cognitive complaints do not increase the risk of AD. On the contrary, 4.25% of the sample (13 subjects) constantly showed low ACD (i.e., self-report < informant-report) and had significantly higher amyloid burden than the reference class.

The transition from heightened ACD or hypernosognosia (patient report > informant report or test scores) to accurate ACD (patient report = informant report or test scores) and finally anosognosia (patient report < informant report or test scores) is gradual as does the accumulation of brain damage and disease progression. There are few studies to date that have attempted to establish at what moment of the course of the disease the patient no longer complains more than the informant and at what moment this begins to represent a real anosognosia. According to Hanseeuw et al. (2020), patients are no longer hypernosognosic about a year and a half before the diagnosis of MCI and that the onset of frank anosognosia begins during the prodromal phase (just over 3 years before the diagnosis of dementia). In our meta-analysis, MCI subjects had poorer ACD than healthy controls, but higher ACD than subjects with mild dementia. This suggests that in the prodromal phase of the disease, anosognosia is already present, although in a milder form than in the dementia stage. These results are very important when considering that subjective cognitive decline is a criterion for the diagnosis of MCI. This may actually contribute to misdiagnosis (Edmonds et al., 2014, 2018). On the one hand, this can lead to false-positives (individuals followed up for a suspected AD while their SCD is due to another cause). On the other hand, many individuals who underestimate their decline and are at greater risk of having a neurodegenerative disease may not have an adequate medical follow-up.

Finally, ACD would gradually lead to marked anosognosia in the advanced stage of dementia. Indeed, the widespread brain damage occurring in the advanced stages of AD compromises the information transfer and the anterograde memory, among other functions. Generally, this results in the patients having a very altered perception of their current experience, reduced awareness of what is happening in their surroundings, and to their state of health (O'Shaughnessy et al., 2021). At the late stage of the disease, the degradation is so massive that it affects not only the awareness of being ill but also the self-knowledge and sense of identity (Addis and Tippett, 2004).

Consistent with the reviewed neuroimaging studies, the patients who have anosognosia are those who show more marked damage in prefrontal and temporoparietal regions, and they generally present an amnestic and dysexecutive clinical phenotype, which is the most common clinical presentation of AD. From the analysis of the literature, it appears that anosognosia is due to the dysfunction of a specific network, mainly in the right hemisphere, which includes: (i) prefrontal areas (dorsolateral, anterior cingulate, orbitofrontal), the lesion of which would compromise the online monitoring of performance, error detection and update of self-knowledge; (ii) dorsal and medial temporoparietal regions (e.g., posterior cingulate, angular gyrus, precuneus), which are the substrate of our capacity of judging our own performance assuming a third-person perspective; (iii) medial temporal regions, the dysfunction of which can lead to memory deficits, preventing proper comparison between current and past performance, and in particular causing the patient to judge current performance and abilities by anchoring to pre-morbid abilities.

To sum up, there would be a phase of heightened ACD or *hypernosognosia* at the very beginning of the disease in which the subject expresses cognitive complaints. Then the ACD would begin to progressively decrease leading to anosognosia during the prodromal phase and—especially—during the dementia phase. However, we do not preclude the existence of individual deviations from this model. A large variability may be ascribed to inter-individual differences in clinical phenotype of AD, premorbid personality traits, levels of anxiety, depression and nosophobia, comorbidity, cognitive reserve, and the localization of cerebral damage due to AD, among other factors (Alladi et al., 2006). Individual variability may range from severely decreased ACD since early pathological changes, to preserved ACD throughout the disease, as indicated by the lighter areas on each side of the colored lines of Figure 3.

We also propose a second scenario (Figure 3B), which represents the *worried-well* population, with persistent SCD without evidence of cognitive impairment, and without these subjective difficulties being confirmed by an informant. These individuals do not have an underlying AD pathology. We believe, consistently with Jessen et al. (2020), that confirmation of decline by an informant is one of the most important factors to consider when a patient with cognitive complaints seeks medical advice. This could allow distinguishing those who report cognitive changes due to an underlying neurodegenerative disease from worried-well individuals.

In practice, the clinician should always listen carefully to the patient's complaint. It might start by asking a general question about the reason of the visit. Subsequently, depending on the answer to the first question, the clinician may ask more specific questions, for example “do you happen to have memory difficulties?”, “Are you having trouble finding words?”, etc. This procedure helps to distinguish what the patient perceives as the main problem. It could happen that the patient reports some memory failures, but he or she could attribute them to age, and say that he or she is seeking medical advice because the family insisted. Since cognitive complaints are rather non-specific and present at every age (Dubois and Agid, 2002), the clinician should compare patient's complaints with a more objective source of information. An individual who is seeking medical advice for cognitive problems spontaneously or at the suggestion of his or her family, should perform a neuropsychological assessment to clarify if the perception of decline is confirmed by objective tests. The informant's assessment is also a very important time- and cost-saving source of information that the clinician should always consider. Although informant's assessment may be subject to bias, a tendency to underestimate cognitive decline is more likely to be the result of an ongoing neurodegenerative process (this is most commonly Alzheimer's disease, but not limited to). In research settings, ACD should be systematically measured by including a study-partner or by comparing subjective and objective decline. Another simple and quick method to evaluate the patient's degree of awareness is to ask him or her to evaluate his or her performance on a neuropsychological test just performed. This can be done simply by asking the patient “How do you think you performed this test?” moments after completing it. Or, for a more accurate and reliable measurement, one of the procedures proposed in the literature and described above can be adopted.

The framework and staging schemas described above have been drafted after considering the diversity of previous research findings, and developed to address the need for an integration of the existing evidence.

Further—and particularly longitudinal—studies are needed, to confirm the consecutive presence of hypernosognosia and poor ACD in the pre-dementia stages. Further studies should consider awareness of illness as a biopsychosocial construct, as many neuropathological, psychoaffective, relational and cognitive factors are known to affect the expression of this symptom. Therefore, the authors should take this into account when designing studies on anosognosia.

### Limitations

The main limitation of this article is that we have carried out a meta-analysis of only a part of the articles (those conducted on subjects with MCI), as it could add evidence to current knowledge. Unfortunately, it was not possible to conduct a meta-analysis of the articles on preclinical AD.

Second, the articles included were heterogeneous. For example, the index of ACD was computed in many different ways, demonstrating that there is not yet a gold standard for the evaluation of ACD.

Third, the pre-dementia stages of AD were defined very differently in the studies, and only a minority of them involved the use of biomarkers to confirm the presence of AD pathology. Similarly, preclinical AD studies have focused on the presence of amyloid to define the condition of an individual at risk. No studies have based the definition of this condition on the simultaneous presence of amyloid and tau. According to certain criteria (for example in Dubois et al., 2016a), a cognitively-intact individual would be at risk of preclinical AD if he/she has a positive pathophysiological marker between amyloid and tau. However, the evidence suggests that the presence of both positive biomarkers increases the specificity of the diagnosis compared to only one of the two (Parnetti et al., 2019). The risk is that the subjects included in the studies discussed above may have cerebral amyloidosis not due to AD, thus making the results found less specific for describing the ACD in AD. The same can also be extended to the studies on MCI.

Forth, although AD has a typical amnestic late-onset clinical manifestation, it is known that atypical forms exist, which are non-amnesic and often of early onset (Gorno-Tempini et al., 2004, 2008). This is the case, for example, of the dysexecutive variant, of the linguistic variant (logopenic primary progressive aphasia) and of posterior variants, for example the visuospatial one. It is also known that the degree of ACD differs in the different variants (Charles and Hillis, 2005). In our review and meta-analysis, we focus on the typical amnesic variant, but we do not exclude the possibility that individuals with different variants may have been included in the study samples.

Fifth, given the paucity of meta-analyzed articles, we decided not to use a certain *p*-value or effect size measure as an additional selection criterion. This may have led to the inclusion of studies reporting small effects.

Finally, we did not include unpublished or gray literature (e.g., dissertations, conference papers) in the review. Indeed, statistically non-significant results are less likely to be published (the so-called “file-drawer problem”), and this could represent a bias and an increased likelihood of Type I errors.

### Conclusion and Implication

The study of ACD since the onset of AD pathology, its evolution and neural correlates is, notably, a piece of the larger understanding of the pre-dementia phase of AD. Therefore, it is a relevant research question in many respects.

First, the presence of poor ACD at the beginning of the disease may delay the search for medical help. Consequently, this limits the possibility of implementing treatment plans, of being included in clinical trials, and potentially delays the access to a disease-modifying treatment when one becomes available.

Furthermore, the lack of ACD is associated with poor decision-making skills (Oba et al., 2019): we might expect the patient with poor ACD to have troubles in making decisions related to his or her health, and in anticipating and preventing potential future work/administrative issues. The patient with reduced ACD may also assume to be able to achieve unrealistic therapeutic goals (for example, a regression of cognitive impairment or the regaining of lost daily-life abilities). The failure of such purposes may generate a sense of frustration, anger, low self-esteem and lack of motivation to continue the treatment.

In addition to this, caregivers' sense of responsibility and commitment may also be higher in the presence of reduced ACD or anosognosia, consequently having more chances to feel depressed and alienated (Starkstein et al., 2010; Spalletta et al., 2012; Jacus et al., 2015; Mak et al., 2015; Jacus, 2017).

Thus, if this symptom is recognized early-on during AD, it might benefit from therapeutic trials specifically targeting poor ACD.

Finally, concerning research, a greater understanding of this symptom could also allow to better describe preclinical and prodromal AD, and could guide researchers to include subjects with poor ACD together with a study-partner in clinical trials and cohort studies.

## Data Availability Statement

The original contributions presented in the study are included in the present article. The data supporting the findings of this study and further inquiries are available from the corresponding author, Federica Cacciamani: federica.cacciamani@icm-institute.org.

## Author Contributions

All authors contributed to the study design and provided expertise and insights into the interpretation of the results. Data were extracted from FC, GG, and MH. MH provided statistical expertise for the meta-analysis. The manuscript was drafted by FC and SE and critically reviewed and approved by all authors.

## Conflict of Interest

BD has received consultancy fees from *Biogen, Boehringer Ingelheim, Eli Lilly*, and *MedAvante* and grants for his institution from *Merck, Pfizer*, and *Roche*. JM has received honoraria, as an educational speaker or consultant from *Roche diagnostics, Genentech, Novartis, Lundbeck, Oryzon, Biogen, Lilly, Janssen, Green Valley, MSD, Eisai, Alector* and *Biocross*. SE has received honoraria as a speaker or consultant for *Eli Lilly, Biogen, Astellas Pharma, Roche* and *GE Healthcare*. SE reports personal fees from *Biogen, Roche*, and *GE Healthcare*, outside the submitted work. The remaining authors declare that the research was conducted in the absence of any commercial or financial relationships that could be construed as a potential conflict of interest.

## Publisher's Note

All claims expressed in this article are solely those of the authors and do not necessarily represent those of their affiliated organizations, or those of the publisher, the editors and the reviewers. Any product that may be evaluated in this article, or claim that may be made by its manufacturer, is not guaranteed or endorsed by the publisher.

## References

[B1] Abdulrab K. Heun R. (2008). Subjective memory impairment. A review of its definitions indicates the need for a comprehensive set of standardised and validated criteria. Euro. Psychiatry 23, 321–330. 10.1016/j.eurpsy.2008.02.004 18434102

[B2] Addis D. R. Tippett L. J. (2004). Memory of myself: autobiographical memory and identity in Alzheimer's disease. Memory 12, 56–74. 10.1080/09658210244000423 15098621

[B3] Agnew S. K. Morris R. G. (1998). The heterogeneity of anosognosia for memory impairment in Alzheimer's disease: a review of the literature and a proposed model. Aging Mental Health 2, 9–15. 10.1080/13607869856876

[B4] Akai T. Hanyu H. Sakurai H. Sato T. Iwamoto T. (2009). Longitudinal patterns of unawareness of memory deficits in mild Alzheimer's disease. Geriatrics Gerontol. Int. 9, 16–20. 10.1111/j.1447-0594.2008.00512.x 19260975

[B5] Alladi S. Arnold R. Mitchell J. Nestor P. J. Hodges J. R. (2006). Mild cognitive impairment: applicability of research criteria in a memory clinic and characterization of cognitive profile. Psychol. Med. 36, 507–515. 10.1017/S0033291705006744 16426486

[B6] Amanzio M. Vase L. Leotta D. Miceli R. Palermo S. Geminiani G. (2013). Impaired awareness of deficits in Alzheimer's disease: the role of everyday executive dysfunction. J. Int. Neuropsychol. Soc. 19, 63–72. 10.1017/S1355617712000896 22995647

[B7] Ansell E. L. Bucks R. S. (2006). Mnemonic anosognosia in Alzheimer's disease: a test of agnew and morris (1998). Neuropsychologia 44, 1095–1102. 10.1016/j.neuropsychologia.2005.10.019 16324727

[B8] Babinski J. (1914). Contribution to the study of the mental disorders in hemiplegia of organic cerebral origin (anosognosia). Translated from the original ≪ Contribution à l'étude des troubles mentaux dans l'hémiplégie organique cérébrale (anosognosie) ≫. Cortex 61, 5–8. 25481462 10.1016/j.cortex.2014.04.019

[B9] Bertrand J. M. Mazancieux A. Moulin C. J. A. Béjot Y. Rouaud O. Souchay C. (2019). In the here and now: short term memory predictions are preserved in Alzheimer's disease. Cortex 119, 158–164. 10.1016/j.cortex.2019.03.027 31132694

[B10] Borenstein M. Hedges L. V. Higgins J. P. T. Rothstein H. R. (2009). Introduction to Meta-Analysis. New York, NY: John Wiley and Sons.

[B11] Cacchione P. Z. Powlishta K. K. Grant E. A. Buckles V. D. Morris J. C. (2003). Accuracy of collateral source reports in very mild to mild dementia of the Alzheimer type. J. Am. Geriatr. Soc. 51, 819–823. 10.1046/j.1365-2389.2003.51263.x 12757569

[B12] Cacciamani F. Sambati L. Houot M. Habert M. O. Dubois B. Epelbaum S.. (2020). Awareness of cognitive decline trajectories in asymptomatic individuals at risk for AD. Alzheimer's Res. Ther. 12:129. 10.1186/s13195-020-00700-8 33054821 PMC7557018

[B13] Cacciamani F. Tandetnik C. Gagliardi G. Bertin H. Habert M. O. Hampel H.. (2017). Low cognitive awareness, but not complaint, is a good marker of preclinical Alzheimer's disease. J. Alzheimers Dis. 59, 753–762. 10.3233/JAD-170399 28671134

[B14] Cations M. Radisic G. Crotty M. Laver K. E. (2018). What does the general public understand about prevention and treatment of dementia? A systematic review of population-based surveys. PLoS ONE 13:e0196085. 10.1371/journal.pone.0196085 29672559 PMC5908164

[B15] Charles R. F. Hillis A. E. (2005). Posterior cortical atrophy: clinical presentation and cognitive deficits compared to Alzheimer's disease. Behav. Neurol. 16, 15–23. 10.1155/2005/762569 16082076 PMC5478852

[B16] Cines S. Farrell M. Steffener J. Sullo L. Huey E. Karlawish J.. (2015). Examining the pathways between self-awareness and well-being in mild to moderate Alzheimer disease. Am. J. Geriatric Psychiatry 23, 1297–1306. 10.1016/j.jagp.2015.05.005 26560509 PMC4653086

[B17] Comijs H. C. Deeg D. J. Dik M. G. Twisk J. W. Jonker C. (2002). Memory complaints; the association with psycho-affective and health problems and the role of personality characteristics. A 6-year follow-up study. J. Affective Disord. 72, 157–165. 10.1016/S0165-0327(01)00453-0 12200206

[B18] Conde-Sala J. L. Reñé-Ramírez R. Turró-Garriga O. Gascón-Bayarri J. Campdelacreu-Fumad,ó J. Juncadella-Puig M.. (2014). Severity of dementia, anosognosia, and depression in relation to the quality of life of patients with Alzheimer disease: discrepancies between patients and caregivers. Am. J. Geriatric Psychiatry 22, 138–147. 10.1016/j.jagp.2012.07.001 23567444

[B19] Conde-Sala J. L. Reñé-Ramírez R. Turró-Garriga O. Gascón-Bayarri J. Juncadella-Puig M. Moreno-Cordón L.. (2013). Clinical differences in patients with Alzheimer's disease according to the presence or absence of anosognosia: implications for perceived quality of life. J. Alzheimer Dis. 33, 1105–1116. 10.3233/JAD-2012-121360 23128559

[B20] Coutinho G. Drummond C. Teldeschi A. Mattos P. (2016). Awareness of memory deficits is useful to distinguish between depression and mild cognitive impairment in the elderly. Rev. Brasileira Psiquiatria. 38, 231–234. 10.1590/1516-4446-2015-1772 27192215 PMC7194276

[B21] Cova I. Grande G. Cucumo V. Ghiretti R. Maggiore L. Galimberti D.. (2017). Self-awareness for memory impairment in amnestic mild cognitive impairment: a longitudinal study. Am. J. Alzheimer Dis Other Dement. 32, 401–407. 10.1177/1533317517725812 28840743 PMC10852863

[B22] Dalla Barba G. La Corte V. Dubois B. (2015). For a cognitive model of subjective memory awareness. J. Alzheimers Dis. 48(Suppl. 1), S57–S61. 10.3233/JAD-150141 26402084

[B23] Dourado M. Marinho V. Soares C. Engelhardt E. Laks J. (2007). Awareness of disease in dementia: development of a multidimensional rating scale. Dementia Neuropsychol. 1, 74–80. 10.1590/S1980-57642008DN10100012 29213371 PMC5619387

[B24] Dourado M. C. Mograbi D. C. Santos R. L. Sousa M. F. Nogueira M. L. Belfort T.. (2014). Awareness of disease in dementia: factor structure of the assessment scale of psychosocial impact of the diagnosis of dementia. J. Alzheimer Dis. 41, 947–956. 10.3233/JAD-140183 24718103

[B25] Dubois B. Agid Y. (2002). Plainte mnésique, trouble cognitif léger et maladie d'Alzheimer au stade prédémentiel, in Vulnérabilité et vieillissement: comment les prévenir, les retarder ou les métriser?, ed Elsevier SAS (Paris: Editions Scientifiques et Médicales), 108–114.

[B26] Dubois B. Epelbaum S. Nyasse F. Bakardjian H. Gagliardi G. Uspenskaya O.. (2018). Cognitive and neuroimaging features and brain β-amyloidosis in individuals at risk of Alzheimer's disease (INSIGHT-preAD): a longitudinal observational study. Lancet Neurol. 17, 335–346. 10.1016/S1474-4422(18)30029-2 29500152

[B27] Dubois B. Feldman H. H. Jacova C. Cummings J. L. Dekosky S. T. Barberger-Gateau P.. (2010). Revising the definition of Alzheimer's disease: a new lexicon. Lancet Neurol. 9, 1118–1127. 10.1016/S1474-4422(10)70223-4 20934914

[B28] Dubois B. Hampel H. Feldman H. H. Scheltens P. Aisen P. Andrieu S.. (2016a). Preclinical Alzheimer's disease: definition, natural history, and diagnostic criteria. Alzheimers Dement. 12, 292–323. 10.1016/j.jalz.2016.02.002 27012484 PMC6417794

[B29] Dubois B. Padovani A. Scheltens P. Rossi A. Dell'Agnello G. (2016b). Timely diagnosis for Alzheimer's disease: a literature review on benefits and challenges. J. Alzheimers Dis. 49, 617–631. 10.3233/JAD-150692 26484931 PMC4927869

[B30] Duke L. M. Seltzer B. Seltzer J. E. Vasterling J. J. (2002). Cognitive components of deficit awareness in Alzheimer's disease. Neuropsychology 16, 359–369. 10.1037/0894-4105.16.3.359 12146683

[B31] Edmonds E. C. Delano-Wood L. Galasko D. R. Salmon D. P. Bondi M. W. Alzheimer's Disease Neuroimaging Initiative (2014). Subjective cognitive complaints contribute to misdiagnosis of mild cognitive impairment. J. Int. Neuropsychol. Soc. 20, 836–847. 10.1017/S135561771400068X 25156329 PMC4172502

[B32] Edmonds E. C. Weigand A. J. Thomas K. R. Eppig J. Delano-Wood L. Galasko D. R.. (2018). Increasing inaccuracy of self-reported subjective cognitive complaints over 24 months in empirically-derived subtypes of mild cognitive impairment *J. Int. Neuropsychol. Soc.* 24, 842–853. 10.1017/S1355617718000486 PMC6173206 30278855

[B33] Farias S. T. Mungas D. Reed B. R. Cahn-Weiner D. Jagust W. Baynes K.. (2008). The measurement of everyday cognition (ECog): scale development and psychometric properties. Neuropsychology 22, 531–544. 10.1037/0894-4105.22.4.531 18590364 PMC2877034

[B34] Ford A. H. Almeida O. P. Flicker L. Garrido G. J. Greenop K. R. Foster J. K.. (2014). Grey matter changes associated with deficit awareness in mild cognitive impairment: a voxel-based morphometry study. J. Alzheimer Dis. 42, 1251–1259. 10.3233/JAD-132678 25024333

[B35] Fujimoto H. Matsuoka T. Kato Y. Shibata K. Nakamura K. Yamada K.. (2017). Brain regions associated with anosognosia for memory disturbance in Alzheimer's disease: a magnetic resonance imaging study. Neuropsychiatr. Dis. Treat. 13, 1753–1759. 10.2147/NDT.S139177 28740390 PMC5505610

[B36] Gagliardi G. Houot M. Cacciamani F. Habert M. O. Dubois B. Epelbaum S. (2020). The meta-memory ratio: a new cohort-independent way to measure cognitive awareness in asymptomatic individuals at risk for Alzheimer's disease. Alzheimer Res. Therapy 12:57. 10.1186/s13195-020-00626-1 32408882 PMC7222501

[B37] Galeone F. Pappalardo S. Chieffi S. Iavarone A. Carlomagno S. (2011). Anosognosia for memory deficit in amnestic mild cognitive impairment and Alzheimer's disease. Int. J. Geriatr. Psychiatry 26, 695–701. 10.1002/gps.2583 21495076

[B38] Gallo D. A. Chen J. M. Wiseman A. L. Schacter D. L. Budson A. E. (2007). Retrieval monitoring and anosognosia in Alzheimer's disease. Neuropsychology 21, 559–568. 10.1037/0894-4105.21.5.559 17784804

[B39] Garcia-Ptacek S. Cavallin L. Kåreholt I. Kramberger M. G. Winblad B. Jelic V.. (2014). Subjective cognitive impairment subjects in our clinical practice. Dement. Geriatr. Cogn. Dis. Extra 4, 419–430. 10.1159/000366270 25538726 PMC4264484

[B40] Gatchel J. R. Lopera F. Norton D. J. Baena A. Guzman-Velez E. Sanchez J. S.. (2020). Association of subjective cognitive decline with markers of brain pathology in preclinical autosomal dominant Alzheimer's disease. J. Neurol. Neurosurg. Psychiatr. 91, 330–332. 10.1136/jnnp-2019-321205 31874859 PMC7397724

[B41] Gil N. Josman N. (2001). Memory and metamemory performance in Alzheimer's disease and healthy elderly: the contextual memory test (CMT). Aging 13, 309–315. 10.1007/BF03353427 11695500

[B42] Gilleen J. Greenwood K. David A. S. (2014). The role of memory in awareness of memory deficits in Alzheimer's disease, schizophrenia, and brain injury. J. Clin. Exp. Neuropsychol. 36, 43–57. 10.1080/13803395.2013.863835 24344744

[B43] Gorno-Tempini M. L. Brambati S. M. Ginex V. Ogar J. Dronkers N. F. Marcone A.. (2008). The logopenic/phonological variant of primary progressive aphasia. Neurology 71, 1227–1234. 10.1212/01.wnl.0000320506.79811.da 18633132 PMC2676989

[B44] Gorno-Tempini M. L. Dronkers N. F. Rankin K. P. Ogar J. M. Phengrasamy L. Rosen H. J.. (2004). Cognition and anatomy in three variants of primary progressive aphasia. Ann. Neurol. 55, 335–346. 10.1002/ana.10825 14991811 PMC2362399

[B45] Graham D. P. Kunik M. E. Doody R. Snow A. L. (2005). Self-reported awareness of performance in dementia. Brain Res. Cogn. Brain Res. 25, 144–152. 10.1016/j.cogbrainres.2005.05.001 15919186

[B46] Hannesdottir K. Morris R. G. (2007). Primary and secondary anosognosia for memory impairment in patients with Alzheimer's disease. Cortex 43, 1020–1030. 10.1016/S0010-9452(08)70698-1 17941357

[B47] Hanseeuw B. J. Scott M. R. Sikkes S. Properzi M. Gatchel J. R. Salmon E.. (2020). Evolution of anosognosia in alzheimer's disease and its relationship to amyloid. Ann. Neurol. 87, 267–280. 10.1002/ana.25649 31750553 PMC6980336

[B48] Hanyu H. Sato T. Akai T. Shimizu S. Hirao K. Kanetaka H.. (2008). Neuroanatomical correlates of unawareness of memory deficits in early Alzheimer's disease. Dement. Geriatr. Cogn. Disord. 25, 347–353. 10.1159/000119594 18319600

[B49] Harwood D. G. Sultzer D. L. Feil D. Monserratt L. Freedman E. Mandelkern M. A. (2005). Frontal lobe hypometabolism and impaired insight in Alzheimer disease. Am. J. Geriatric Psychiatry 13, 934–941. 10.1097/00019442-200511000-00003 16286436

[B50] Horning S. M. Melrose R. Sultzer D. (2014). Insight in Alzheimer's disease and its relation to psychiatric and behavioral disturbances. Int. J. Geriatr. Psychiatry 29, 77–84. 10.1002/gps.3972 23671016 PMC3796120

[B51] Hurt C. S. Banerjee S. Tunnard C. Whitehead D. L. Tsolaki P. Mecocci P.. (2010). Insight, cognition and quality of life in Alzheimer's disease. J. Neurol. Neurosurg. Psychiatr. 81, 331–336. 10.1136/jnnp.2009.184598 19828481

[B52] Jack C. Bennett D. A. Blennow K. Carrillo M. C. Dunn B. Haeberlein S. B.. (2018). NIA-AA research framework: toward a biological definition of Alzheimer's disease *Alzheimer's Dementia* 14, 535–562. 10.1016/j.jalz.2018.02.018 PMC5958625 29653606

[B53] Jacus J. P. (2017). Awareness, apathy, and depression in Alzheimer's disease and mild cognitive impairment. Brain Behav. 7:e00661. 10.1002/brb3.661 28413709 PMC5390841

[B54] Jacus J. P. Belorgey N. Trivalle C. Gély-Nargeot M. C. (2015). Factors associated with awareness in early stages of Alzheimer's disease and in mild cognitive impairment. Encephale 1, S44–S48. 10.1016/j.encep.2014.08.011 25238907

[B55] Jedidi H. Feyers D. Collette F. Bahri M. A. Jaspar M. d'Argembeau A.. (2014). Dorsomedial prefrontal metabolism and unawareness of current characteristics of personality traits in Alzheimer's disease. Soc. Cogn. Affect. Neurosci. 9, 1458–1463. 10.1093/scan/nst132 23946004 PMC4187259

[B56] Jessen F. Amariglio R. E. Buckley R. F. van der Flier W. M. Han Y. Luis Molinuevo J.. (2020). The characterisation of subjective cognitive decline. Lancet Neurol. 19, 271–278. 10.1016/S1474-4422(19)30368-0 31958406 PMC7062546

[B57] Jessen F. Amariglio R. E. van Boxtel M. Breteler M. Ceccaldi M. Chetelat G.. (2014). A conceptual framework for research on subjective cognitive decline in preclinical Alzheimer's disease. Alzheimer's Dementia 10, 844–852. 10.1016/j.jalz.2014.01.001 24798886 PMC4317324

[B58] Kalenzaga S. Clarys D. (2013). Relationship between memory disorders and self-consciousness in Alzheimer's disease. Gériatrie Et Psychologie Neuropsychiatrie Du Vieillissement 11, 187–196. 10.1684/pnv.2013.0403 23803636

[B59] Kashiwa Y. Kitabayashi Y. Narumoto J. Nakamura K. Ueda H. Fukui K. (2005). Anosognosia in Alzheimer's disease: association with patient characteristics, psychiatric symptoms and cognitive deficits. Psychiatry Clin. Neurosci. 59, 697–704. 10.1111/j.1440-1819.2005.01439.x 16401246

[B60] Lacerda I. B. Santos R. L. Belfort T. Neto J. Dourado M. (2020). Patterns of discrepancies in different objects of awareness in mild and moderate Alzheimer's disease. Aging Ment. Health 24, 789–796. 10.1080/13607863.2018.1544219 30474400

[B61] Langer K. G. Levine D. N. (2014). Babinski, J. (1914). Contribution to the study of the mental disorders in hemiplegia of organic cerebral origin (anosognosia). Translated by K.G. Langer and D.N. Levine: Translated from the original Contribution à l'Étude des Troubles Mentaux dans l'Hémiplégie Organique Cérébrale (Anosognosie). Cortex 61, 5–8. 10.1016/j.cortex.2014.04.019 25481462

[B62] Lopez O. L. Becker J. T. Somsak D. Dew M. A. DeKosky S. T. (1994). Awareness of cognitive deficits and anosognosia in probable Alzheimer's disease. Eur. Neurol. 34, 277–282. 10.1159/000117056 7995303

[B63] Mak E. Chin R. Ng L. T. Yeo D. Hameed S. (2015). Clinical associations of anosognosia in mild cognitive impairment and Alzheimer's disease. Int. J. Geriatr. Psychiatry 30, 1207–1214. 10.1002/gps.4275 25754519

[B64] Maki Y. Yamaguchia T. Yamaguchia H. (2013). Evaluation of anosognosia in Alzheimer's disease using the symptoms of early dementia-11 questionnaire (SED-11Q). Dement. Geriatr. Cogn. Disord. 3, 351–359. 10.1159/000355367 24403907 PMC3884202

[B65] McGlynn S. M. Kaszniak A. W. (1991). When metacognition fails: impaired awareness of deficit in Alzheimer's disease. J. Cogn. Neurosci. 3, 183–187. 10.1162/jocn.1991.3.2.183 23972092

[B66] McGlynn S. M. Schacter D. L. (1989). Unawareness of deficits in neuropsychological syndromes. J. Clin. Exp. Neuropsychol. 11, 143–205. 10.1080/01688638908400882 2647781

[B67] Migliorelli R. Tesón A. Sabe L. Petracca G. Petracchi M. Leiguarda R.. (1995). Anosognosia in Alzheimer's disease: a study of associated factors. J. Neuropsychiatry Clin. Neurosci. 7, 338–344. 7580194 10.1176/jnp.7.3.338

[B68] Millenaar J. Hvidsten L. de Vugt M. E. Engedal K. Selbæk G. Wyller T. B.. (2017). Determinants of quality of life in young onset dementia - results from a European multicenter assessment. Aging Ment. Health. 21, 24–30. 10.1080/13607863.2016.1232369 27676211

[B69] Mimura M. Yano M. (2006). Memory impairment and awareness of memory deficits in early-stage Alzheimer's disease. Rev. Neurosci. 1, 253–266. 10.1515/REVNEURO.2006.17.1-2.253 16703956

[B70] Mograbi D. C. Brown R. G. Morris R. G. (2009). Anosognosia in Alzheimer's disease-the petrified self. Conscious. Cogn. 18, 989–1003. 10.1016/j.concog.2009.07.005 19683461

[B71] Mograbi D. C. Brown R. G. Salas C. Morris R. G. (2012). Emotional reactivity and awareness of task performance in Alzheimer's disease. Neuropsychologia 50, 2075–2084. 10.1016/j.neuropsychologia.2012.05.008 22609573

[B72] Molinuevo J. L. Gramunt N. Gispert J. D. Fauria K. Esteller M. Minguillon C.. (2016). The ALFA project: a research platform to identify early pathophysiological features of Alzheimer's disease. Alzheimer's Dementia 2, 82–92. 10.1016/j.trci.2016.02.003 29067295 PMC5644283

[B73] Monahan P. O. Alder C. A. Khan B. A. Stump T. Boustani M. A. (2014). The healthy aging brain care (HABC) monitor: validation of the patient self-report version of the clinical tool designed to measure and monitor cognitive, functional, and psychological health. Clin. Interv. Aging 9, 2123–2132. 10.2147/CIA.S64140 25584024 PMC4264599

[B74] Monahan P. O. Boustani M. A. Alder C. Galvin J. E. Perkins A. J. Healey P.. (2012). Practical clinical tool to monitor dementia symptoms: the HABC-monitor. Clin. Interv. Aging 7, 143–157. 10.2147/CIA.S30663 22791987 PMC3393358

[B75] Mosconi L. Pupi A. De Leon M. J. (2008). Brain glucose hypometabolism and oxidative stress in preclinical Alzheimer's disease. Ann. N. Y. Acad. Sci. 1147, 180–195. 10.1196/annals.1427.007 19076441 PMC2661241

[B76] Munro C. E. Donovan N. J. Amariglio R. E. Papp K. V. Marshall G. A. Rentz D. M.. (2018). The impact of awareness of and concern about memory performance on the prediction of progression from mild cognitive impairment to Alzheimer disease dementia. Am. J. Geriatric Psychiatry 26, 896–904. 10.1016/j.jagp.2018.04.008 29866588 PMC6959130

[B77] Nobili F. Mazzei D. Dessi B. Morbelli S. Brugnolo A. Barbieri P.. (2010). Unawareness of memory deficit in amnestic MCI: FDG-PET findings. J. Alzheimer Dis. 22, 993–1003. 10.3233/JAD-2010-100423 20858977

[B78] Oba H. Matsuoka T. Imai A. Fujimoto H. Kato Y. Shibata K.. (2019). Interaction between memory impairment and depressive symptoms can exacerbate anosognosia: a comparison of Alzheimer's disease with mild cognitive impairment. Aging Mental Health 23, 595–601. 10.1080/13607863.2018.1442411 29528693

[B79] O'Connell M. E. Dal Bello-Haas V. Crossley M. Morgan D. (2014). Clinical correlates of awareness for balance, function, and memory: evidence for the modality specificity of awareness. J. Aging Res. 1, 674–716. 10.1155/2014/674716 24551452 PMC3914567

[B80] Onor M. L. Trevisiol M. Negro C. Aguglia E. (2006). Different perception of cognitive impairment, behavioral disturbances, and functional disabilities between persons with mild cognitive impairment and mild Alzheimer's disease and their caregivers. Am. J. Alzheimer Dis Other Demen. 21, 333–338. 10.1177/1533317506292454 17062552 PMC10832643

[B81] Orfei M. D. Varsi A. E. Blundo C. Celia E. Casini A. R. Caltagirone C.. (2010). Anosognosia in mild cognitive impairment and mild Alzheimer's disease: frequency and neuropsychological correlates. Am. J. Geriatric Psychiatry 18, 1133–1140. 10.1097/JGP.0b013e3181dd1c50 20808100

[B82] O'Shaughnessy N. J. Chan J. E. Bhome R. Gallagher P. Zhang H. Clare L.. (2021). Awareness in severe Alzheimer's disease: a systematic review. Aging Mental Health 25, 602–612. 10.1080/13607863.2020.1711859 31942805

[B83] Oyebode J. R. Telling A. L. Hardy R. M. Austin J. (2007). Awareness of memory functioning in early Alzheimer's disease: lessons from a comparison with healthy older people and young adults. Aging Ment. Health 11, 761–767. 10.1080/13607860701366277 18074264

[B84] Parnetti L. Chipi E. Salvadori N. D'Andrea K. Eusebi P. (2019). Prevalence and risk of progression of preclinical Alzheimer's disease stages: a systematic review and meta-analysis. Alzheimer Res. Ther. 11:7. 10.1186/s13195-018-0459-7 30646955 PMC6334406

[B85] Prigatano G. P. (1987). Personality and psychosocial consequences after brain injury, in Neuropsychological Rehabilitation, eds Meir M. Dillard L. Benton A. (London: Churchill Livingston).

[B86] Rabin L. A. Smart C. M. Crane P. K. Amariglio R. E. Berman L. M. Boada M.. (2015). Subjective cognitive decline in older adults: an overview of self-report measures used across 19 international research studies. J. Alzheimer Dis. 48(Suppl. 1), S63–S86. 10.3233/JAD-150154 26402085 PMC4617342

[B87] Rattanabannakit C. Risacher S. L. Gao S. Lane K. A. Brown S. A. McDonald B. C.. (2016). The cognitive change index as a measure of self and informant perception of cognitive decline: relation to neuropsychological tests. J. Alzheimer Dis. 51, 1145–1155. 10.3233/JAD-150729 26923008 PMC4833578

[B88] Ries M. L. Jabbar B. M. Schmitz T. Trivedi M. A. Gleason C. E. Carlsson C. M.. (2007). Anosognosia in mild cognitive impairment: relationship to activation of cortical midline structures involved in self-appraisal. J. Int. Neuropsychol. Soc. 13, 450–461. 10.1017/S1355617707070488 17445294 PMC2654607

[B89] Risacher S. L. McDonald B. C. Tallman E. F. West J. D. Farlow M. R. Unverzagt F. W.. (2016). Association between anticholinergic medication use and cognition, brain metabolism, and brain atrophy in cognitively normal older adults. JAMA Neurol. 73, 721–732. 10.1001/jamaneurol.2016.0580 27088965 PMC5029278

[B90] Robins J. Breslow N. Greenland S. (1986). Estimators of the mantel-haenszel variance consistent in both sparse data and large-strata limiting models. Biometrics 42, 311–323. 10.2307/2531052 3741973

[B91] Salmon E. Perani D. Herholz K. Marique P. Kalbe E. Holthoff V.. (2006). Neural correlates of anosognosia for cognitive impairment in Alzheimer's disease. Hum. Brain Mapp. 27, 588–597. 10.1002/hbm.20203 16247783 PMC6871369

[B92] Sanchez-Benavides G. Grau-River O. Cacciaglia R. Suarez-Calvet M. Falcon C. Minguillon C.. (2018). Distinct cognitive and brain morphological features in healthy subjects unaware of informant-reported cognitive decline. J. Alzheimer Dis. 65, 181–191. 10.3233/JAD-180378 30010134 PMC6087444

[B93] Schacter D. L. (1989). On the relation between memory and consciousness: dissociable interactions and conscious experience, in Varieties of Memory and Consciousness: Essays in Honour of Endel Tulving, eds Roediger H. L. III Craik F. (New York, NY: Psychology Press), 355–389.

[B94] Scherling C. S. Wilkins S. E. Zakrezewski J. Kramer J. H. Miller B. L. Weiner M. W.. (2016). Decreased self-appraisal accuracy on cognitive tests of executive functioning is a predictor of decline in mild cognitive impairment. Front. Aging Neurosci. 8:120. 10.3389/fnagi.2016.00120 27458368 PMC4930951

[B95] Senturk G. Bilgic B. Arslan A. B. Bayram A. Hanagasi H. Gurvit H.. (2017). Cognitive and anatomical correlates of anosognosia in amnestic mild cognitive impairment and early-stage Alzheimer's disease. Int. Psychogeriatr. 29, 293–302. 10.1017/S1041610216001812 27780496

[B96] Silva A. Pinho M. S. Macedo L. Souchay C. Moulin C. (2017). Mnemonic anosognosia in Alzheimer's disease is caused by a failure to transfer online evaluations of performance: evidence from memory training programs. J. Clin. Exp. Neuropsychol. 39, 419–433. 10.1080/13803395.2016.1231799 27677926

[B97] Slot R. Verfaillie S. Overbeek J. M. Timmers T. Wesselman L. Teunissen C. E.. (2018). Subjective cognitive impairment cohort (SCIENCe): study design and first results. Alzheimers Res. Ther. 10, 1–13. 10.1186/s13195-018-0390-y 30081935 PMC6080529

[B98] Spalletta G. Girardi P. Caltagirone C. Orfei M. D. (2012). Anosognosia and neuropsychiatric symptoms and disorders in mild Alzheimer disease and mild cognitive impairment. J. Alzheimer Dis. 29, 761–772. 10.3233/JAD-2012-111886 22349686

[B99] Starkstein S. E. Brockman S. Bruce D. Petracca G. (2010). Anosognosia is a significant predictor of apathy in Alzheimer's disease. J. Neuropsychiatry Clin. Neurosci. 22, 378–383. 10.1176/jnp.2010.22.4.378 21037121

[B100] Stewart G. McGeown W. J. Shanks M. F. Venneri A. (2010). Anosognosia for memory impairment in Alzheimer's disease. Acta Neuropsychiatr. 22, 180–187. 10.1111/j.1601-5215.2010.00463.x 25385123

[B101] Stites S. D. Karlawish J. Harkins K. Rubright J. D. Wolk D. (2017). Awareness of mild cognitive impairment and mild Alzheimer's disease dementia diagnoses associated with lower self-ratings of quality of life in older adults. J. Gerontol. Ser. B Psychol. Sci. Soc. Sci. 72, 974–985. 10.1093/geronb/gbx100 28958089 PMC5927082

[B102] Tariot P. N. Lopera F. Langbaum J. B. Thomas R. G. Hendrix S. Schneider L. S.. (2018). The Alzheimer's prevention initiative autosomal-dominant Alzheimer's disease trial: a study of crenezumab versus placebo in preclinical PSEN1 E280A mutation carriers to evaluate efficacy and safety in the treatment of autosomal-dominant Alzheimer's disease, including a placebo-treated noncarrier cohort. Alzheimer's Dementia 4, 150–160. 10.1016/j.trci.2018.02.002 29955659 PMC6021543

[B103] Therriault J. Ng K. P. Pascoal T. A. Mathotaarachchi S. Kang M. S. Struyfs H.. (2018). Anosognosia predicts default mode network hypometabolism and clinical progression to dementia. Neurology 90, e932–e939. 10.1212/WNL.0000000000005120 29444971 PMC5858945

[B104] Tondelli M. Barbarulo A. M. Vinceti G. Vincenzi C. Chiari A. Nichelli P. F.. (2018). Neural correlates of anosognosia in Alzheimer's disease and mild cognitive impairment: a multi-method assessment. Front. Behav. Neurosci. 17:100. 10.3389/fnbeh.2018.00100 29867398 PMC5966556

[B105] Tremont G. Alosco M. L. (2011). Relationship between cognition and awareness of deficit in mild cognitive impairment. Int. J. Geriatr. Psychiatry 26, 299–306. 10.1002/gps.2529 20623477

[B106] Trigg R. Watts S. Jones R. Tod A. (2011). Predictors of quality of life ratings from persons with dementia: the role of insight. Int. J. Geriatr. Psychiatry 26, 83–91. 10.1002/gps.2494 21157853

[B107] Turró-Garriga O. Garre-Olmo J. Reñé-Ramíre R. Calvó-Perxas L. Gascón-Bayarri J. Conde-Sala J. L. (2016). Consequences of anosognosia on the cost of caregivers' care in Alzheimer's disease. J. Alzheimer Dis. 54, 1551–1560. 10.3233/JAD-160419 27636844

[B108] Vannini P. Amariglio R. Hanseeuw B. Johnson K. A. McLaren D. G. Chhatwal J.. (2017a). Memory self-awareness in the preclinical and prodromal stages of Alzheimer's disease. Neuropsychologia 99, 343–349. 10.1016/j.neuropsychologia.2017.04.002 28385579 PMC5473166

[B109] Vannini P. Hanseeuw B. Munro C. E. Amariglio R. E. Marshall G. A. Rentz D. M.. (2017b). Anosognosia for memory deficits in mild cognitive impairment: insight into the neural mechanism using functional and molecular imaging. NeuroImage Clin. 15, 408–414. 10.1016/j.nicl.2017.05.020 28616381 PMC5458095

[B110] Vannini P. Hanseeuw B. J. Gatchel J. R. Sikkes S. Alzate D. Zuluaga Y.. (2020). Trajectory of unawareness of memory decline in individuals with autosomal dominant Alzheimer disease. JAMA Netw. Open 3:e2027472. 10.1001/jamanetworkopen.2020.27472 33263761 PMC7711319

[B111] Verfaillie S. C. J. Timmers T. Slot R. E. R. van der Weijden C. W. J. Wesselman L. M. P. Prins N. D.. (2019). Amyloid-β Load is related to worries, but not to severity of cognitive complaints in individuals with subjective cognitive decline: the SCIENCe project. Front. Aging Neurosci. 11:7. 10.3389/fnagi.2019.00007 30760996 PMC6362417

[B112] Vermunt L. Sikkes S. van den Hout A. Handels R. Bos I. van der Flier W. M.. (2019). Duration of preclinical, prodromal, and dementia stages of Alzheimer's disease in relation to age, sex, and APOE genotype. Alzheimer Dementia 15, 888–898. 10.1016/j.jalz.2019.04.001 31164314 PMC6646097

[B113] Vogel A. Hasselbalch S. G. Gade A. Ziebell M. Waldemar G. (2005). Cognitive and functional neuroimaging correlates for anosognosia in mild cognitive impairment and Alzheimer's disease. Int. J. Geriatr. Psychiatry 20, 238–246. 10.1002/gps.1272 15717342

[B114] Wilson R. S. Boyle P. A. Yu L. Barnes L. L. Sytsma J. Buchman A. S.. (2015). Temporal course and pathological basis of unawareness of memory loss in dementia. Neurology 18, 984–991. 10.1212/WNL.0000000000001935 26311746 PMC4567465

[B115] Wolfsgruber S. Jessen F. Koppara A. Kleineidam L. Schmidtke K. Frölich L.. (2015). Subjective cognitive decline is related to CSF biomarkers of AD in patients with MCI. Neurology 84, 1261–1268. 10.1212/WNL.0000000000001399 25716354

[B116] Zamboni G. Drazich E. McCulloch E. Filippini N. Mackay C. E. Jenkinson L.. (2013). Neuroanatomy of impaired self-awareness in Alzheimer's disease and mild cognitive impairment. Cortex 49, 668–678. 10.1016/j.cortex.2012.04.011 22677047

