# Supplementary material for: Awareness of Cognitive Decline in Patients With Alzheimer's Disease: A Systematic Review and Meta-Analysis
Source: Front Aging Neurosci. 2021 Aug 3;13:697234. doi: 10.3389/fnagi.2021.697234 (PMC8370471; doi:10.3389/fnagi.2021.697234)

SUPPLEMENTARY MATERIALS

Table S1. Flow diagram for study selection and analysis

Records removed before screening:

Duplicate records removed

(n = 157)

Records identified from:

Pubmed (n = 382)

Scopus (n = 398)

Reference lists (n = 39)

**Identification**

Records screened and assessed for eligibility

(n = 662)

Records excluded because irrelevant for the purpose of the review

(n = 379)

**Screening**

Studies included in the systematic review

(n = 283)

Studies including both subjects with MCI and with AD dementia

(n = 24)

**Included**

Studies only including subjects at risk for AD

(pre-AD)

(n = 4)

Studies only including subjects with AD dementia

(n = 225)

Studies only including subjects with MCI

(n = 26)

Studies including subjects both at risk of AD (pre-AD) and with MCI

(n = 4)

**Systematic review**

Studies included in the meta-analysis

(n = 18)

Studies excluded because they reported neither a continuous measure of ACD (mean and standard deviation), nor the percentage of aware and unaware subjects

(n = 36)

**Meta-analysis**


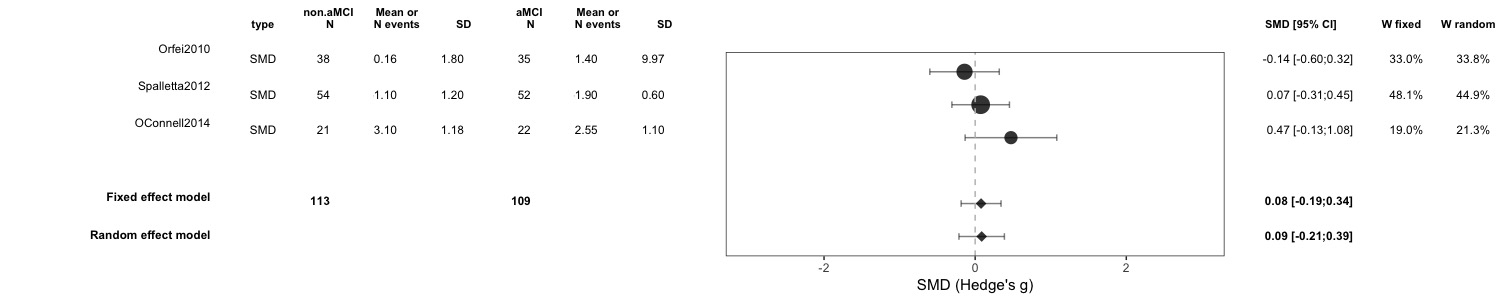
Figure S2. Meta-analysis forest plots.


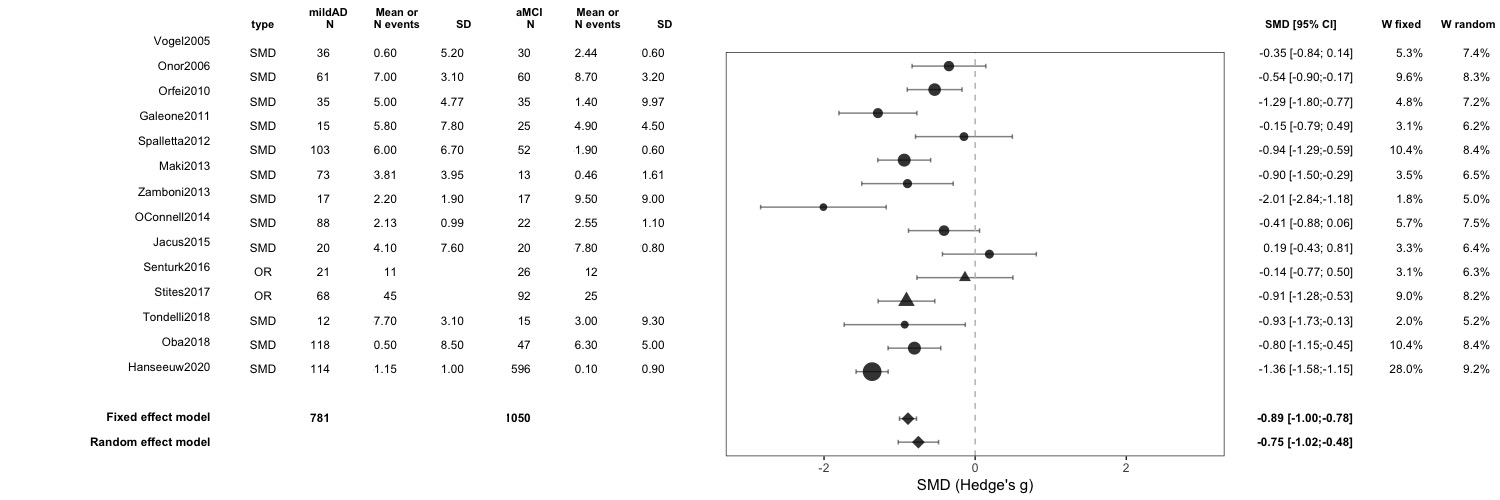


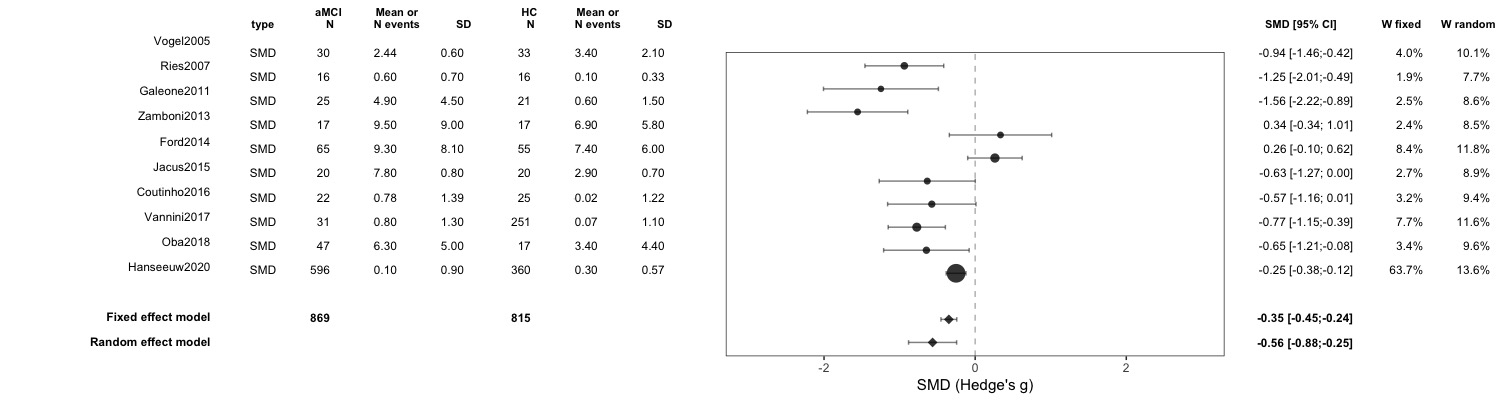


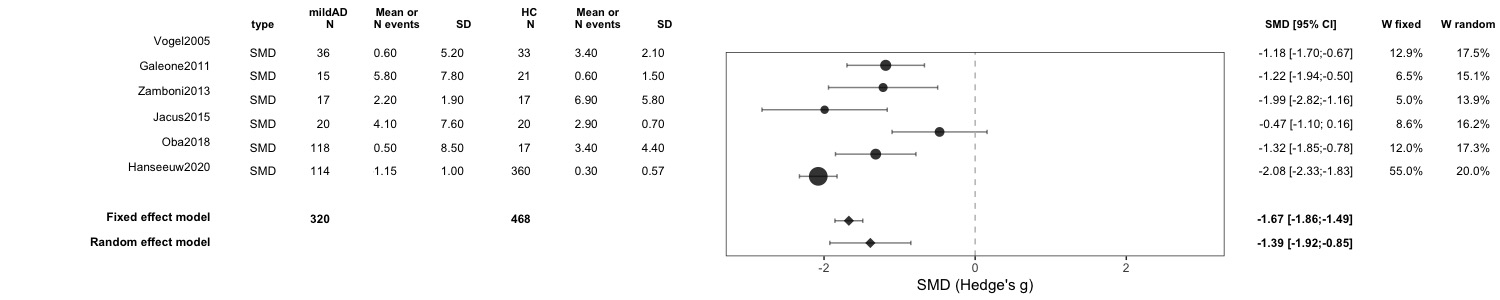


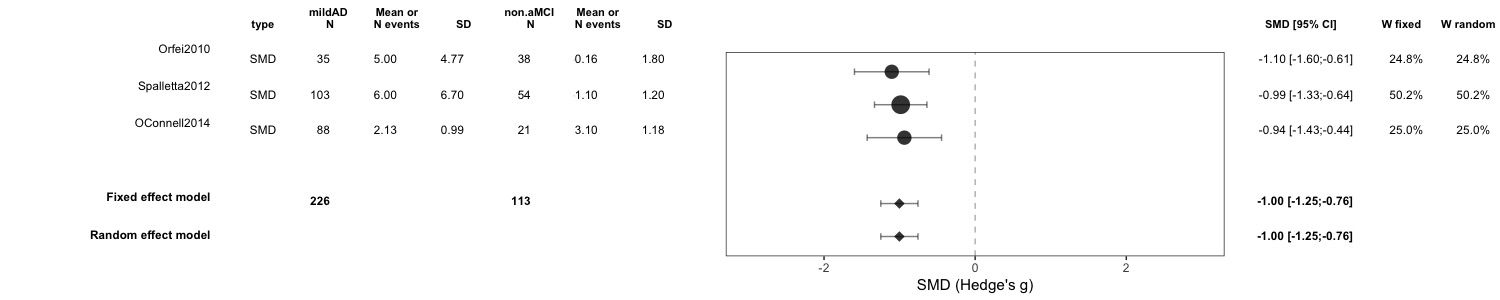

Supplement: Supplementary file 1 [file Data_Sheet_1.docx]
